# Social interactions of eating behaviour among high school students: a cellular automata approach

**DOI:** 10.1186/1471-2288-12-155

**Published:** 2012-10-09

**Authors:** Vahid Dabbaghian, Vijay K Mago, Tiankuang Wu, Charles Fritz, Azadeh Alimadad

**Affiliations:** 1 The Modelling of Complex Social Systems (MoCSSy) Program, The IRMACS Centre, Simon Fraser University, Burnaby, Canada; 2 Department of Mathematics, Simon Fraser University, Burnaby, Canada; 3 Department of Geography, Simon Fraser University, Burnaby, Canada; 4 Faculty of Health Sciences, Simon Fraser University, Burnaby, Canada

## Abstract

**Background:**

Overweight and obesity in children and adolescents is a global epidemic posing problems for both developed and developing nations. The prevalence is particularly alarming in developed nations, such as the United States, where approximately one in three school-aged adolescents (ages 12-19) are overweight or obese. Evidence suggests that weight gain in school-aged adolescents is related to energy imbalance exacerbated by the negative aspects of the school food environment, such as presence of unhealthy food choices. While a well-established connection exists between the food environment, presently there is a lack of studies investigating the impact of the social environment and associated interactions of school-age adolescents. This paper uses a mathematical modelling approach to explore how social interactions among high school adolescents can affect their eating behaviour and food choice.

**Methods:**

In this paper we use a Cellular Automata (CA) modelling approach to explore how social interactions among school-age adolescents can affect eating behaviour, and food choice. Our CA model integrates social influences and transition rules to simulate the way individuals would interact in a social community (e.g., school cafeteria). To replicate these social interactions, we chose the Moore neighbourhood which allows all neighbours (eights cells in a two-dimensional square lattice) to influence the central cell. Our assumption is that individuals belong to any of four states; Bring Healthy, Bring Unhealthy, Purchase Healthy, and Purchase Unhealthy, and will influence each other according to parameter settings and transition rules. Simulations were run to explore how the different states interact under varying parameter settings.

**Results:**

This study, through simulations, illustrates that students will change their eating behaviour from unhealthy to healthy as a result of positive social and environmental influences. In general, there is one common characteristic of changes across time; students with similar eating behaviours tend to form groups, represented by distinct clusters. Transition of healthy and unhealthy eating behaviour is non-linear and a sharp change is observed around a critical point where positive and negative influences are equal.

**Conclusions:**

Conceptualizing the social environment of individuals is a crucial step to increasing our understanding of obesogenic environments of high-school students, and moreover, the general population. Incorporating both contextual, and individual determinants found in real datasets, in our model will greatly enhance calibration of future models. Complex mathematical modelling has a potential to contribute to the way public health data is collected and analyzed.

## Background

Overweight and obesity in children and adolescents is a global epidemic posing problems for both developed and developing nations [1]. The prevalence is particularly alarming in developed nations, such as the United States, where approximately one in three school-aged adolescents (ages 12-19) are overweight or obese [2]. Overweight and obesity in children and adolescents *may increase both* biological and mental health risks, such as hypertension, higher cholesterol [3], low self-esteem and behaviour problems [4]. Addressing these risk factors are especially important as young adults face a more difficult time maintaining healthy weights [5] and physical activity levels [6]. Conceptualizing how the environment can promote or protect individuals from becoming overweight or obese are at the forefront of research in this area, and can provide much needed insight for developing key prevention and intervention strategies [7-10].

For school-age adolescents, obesogenic environments comprise the micro-level influences of home, school, and after-school or social settings and the macro-level influences of school food policies, and food marketing [11]. While a well-established connection exists between both the food and physical activity environments and weight gain, presently there is a lack of studies investigating the impact of the social environment and associated interactions on school-age adolescents. Research has shown that the school food environment may influence the eating behaviour of adolescents through the easy access to unhealthy snacks and the social interactions among peers [12,13]. The influence of peers on one another’s decision making is suggested to be a factor in other health-related behaviours, such as alcohol consumption [14] and smoking [15]. Individual food choice is suspected to ultimately depend on a mixture of environmental, and individual factors. An underlying mechanism suspected to be influential in social situations around eating behaviour is the social facilitation of eating, which posits that individuals are more likely to eat larger quantities when around close friends and relatives [16]. However, other studies have found overweight individuals to eat less when around normal-weight peers, while still consuming more around overweight peers whom they are twice as likely to be friend with [13,17,18].

The finer mechanisms of adolescent social interactions and eating behaviour are less understood. Alteration of one’s eating behaviour is suspected to be caused by weight stigma, and social norms of thinness, sometimes involving the use of unhealthy weight-control behaviours, such as taking diet pills and meal [19,20]. Some studies have found that the type of food eaten by peers, both unhealthy and healthy, affects the personal choice of the individual [21,22]. Understanding the social interactions of adolescents and how they affect eating behaviour and food choice is complex. In order to build effective prevention and intervention strategies, an integrated approach needs to be adopted to explore the multiple associated factors. While this evidence provides broad implications that form a strong foundation for further research, social interactions require a complex conceptualization that has yet to be established.

Recent studies have applied predictive modelling approaches to map the social networks of individuals, finding social interactions to be associated with individual weight gain and obesity [23-25]. By applying a social network model, Bahr et al. [24] found that well-connected actors in a social network had a large impact on neighbouring actors’ body weight. Their study used simulated data that was validated by results from the Framingham Heart Study (FHS). An analysis that used longitudinal data from the FHS also found obesity to be related to social network, estimating the risk of becoming obese would increase by 57% if the person had an obese friend [23]. These studies suggest that a computational modelling approach can effectively analyze multi-faceted problems, as their flexible nature allows multiple scenarios to be tested with varying parameter levels for each variable. A recent review [26] underlined the importance of computational modelling to the epidemiology discipline, where many processes are being unraveled as more dynamic and multi-leveled than previously assumed. The strength of computational models to incorporate dynamically linked variables over time and compare to different outcomes for the same variables is beneficial for a field where outcomes are context and time specific, and potentially dependent on several other variables.

In this study we use a Cellular Automata (CA) modelling approach to explore how social interactions among high school adolescents can affect eating behaviour, and food choice. A CA model assumes a discrete space, resembling a square grid structure, built from a pre-specified amount of uniformly patterned cells. Each cell or square can represent an entity or subject, (e.g., individual, community, idea). The space may represent actual geographic space, or it can represent a population of entities with no geographical relation but a proximal association. Transition rules are assigned to each cell that determine how each cell is assumed to interact with adjacent neighbours and to what degree. Influence from one cell to the other can be adjusted to account for different social scenarios. In our exploratory model, we modeled four types of students who interact in a North American high school setting; (1) students bringing healthy food to school from home, (2) students bringing unhealthy food to school from home, (3) students purchasing healthy food from school vendors, and (4) students purchasing unhealthy food from school vendors. Many studies have examined the social dynamics of the school food environment [27-29] but none have used a computational modelling approach as this paper intends to do. This exploratory model will illustrate the potential for computational modelling to aid in the conceptualization of obesogenic environments of school-age adolescents, and potentially bring light to finer relationships of such processes.

## Methods

### Cellular automata models

Cellular automata is a mathematical modelling technique that can effectively analyze non-linear transmissions of human behaviour among individuals in a real or simulated community. In a CA model the population is represented by two dimensional square grid where each cell indicates an individual in the population. The state of each cell can vary depending on pre-determined rules. These rules are derived from an existing theoretical framework describing a particular phenomenon and are used to model what is happening in the real world. A CA model can effectively capture social interactions that happen over time [30,31]. Since each cell has the capability of holding the information pertaining to that cell, changes can be recorded. In general, CA models measure time discretely, in other words, progress through time is represented as a series of time steps. The cells capture the information at each time step and their states can alter through successive time steps [32].

In order to simplify the complexity of human behaviour, CA modelling must make assumptions which are supported by previous research in this area. In this CA model the underlying premise is that individuals are socially influenced to have healthy or unhealty eating behaviour. As well, the social behaviour of an individual is influenced by others in the community. In this model each cell can potentially be influenced by at most eight surrounding cells.

In a CA model, the change in cells accumulates over time, called time-steps. Each cell has the capability to store its own information from the previous time-step and incorporate that knowledge in future iterations. Cells respond to the new information at each time step and alter their states accordingly in the subsequent time-step. The state of each cell is updated simultaneously at each time step via a pre-determined set of transition rules that govern the neighbourhood interactions; or in this case, social interactions in a school cafeteria.

### Eating behaviour model

Our CA model integrates social influences and transition rules to simulate the way individuals would interact in a social community (e.g., school cafeteria). The cells change over time-steps as they receive and give social influence to their neighbours, updating cell states to reflect the subsequent modifications. Since this is a scenario-based model, the variables can be set according to real input data and adjusted to reflect hypothetical changes in the community.

Although the cells are stationary, the state of the cell can vary. This reflects the change in social state individuals may experience during their life course. These changes occur as a result of social influences and experiences. To replicate these social interactions, we chose the Moore neighbourhood in which the centre cell gets influenced by all its neighbours, i.e., all eight neighbours exert their influences. The cells at the edges are wrapped-around, left and right edges and top and bottom edges. Topologically, this results in a toroidal, or doughnut shape structure which allows corner cells to have same neighbourhood properties as any cell in the CA (i.e., all cells have eight neighbours).

In our model, each cell represents a single student who interacts within their social neighbourhood. For example, a cell can take on any one of four states based on whether a student brings a “brown-bag” lunch or purchases a cafeteria food, and whether a student’s food is healthy or unhealthy. Table 1 shows the four states used in the model.

**Table 1:** Definition of states based on individual eating preference

**Bring Healthy (BH)**	A student who brings healthy food.
**Bring Unhealthy (BU)**	A student who brings unhealthy food.
**Purchase Healthy (PH)**	A student who purchases healthy food.
**Purchase Unhealthy (PU)**	A student who purchases unhealthy food.

### A deterministic model for eating behaviour

**Figure 1:**
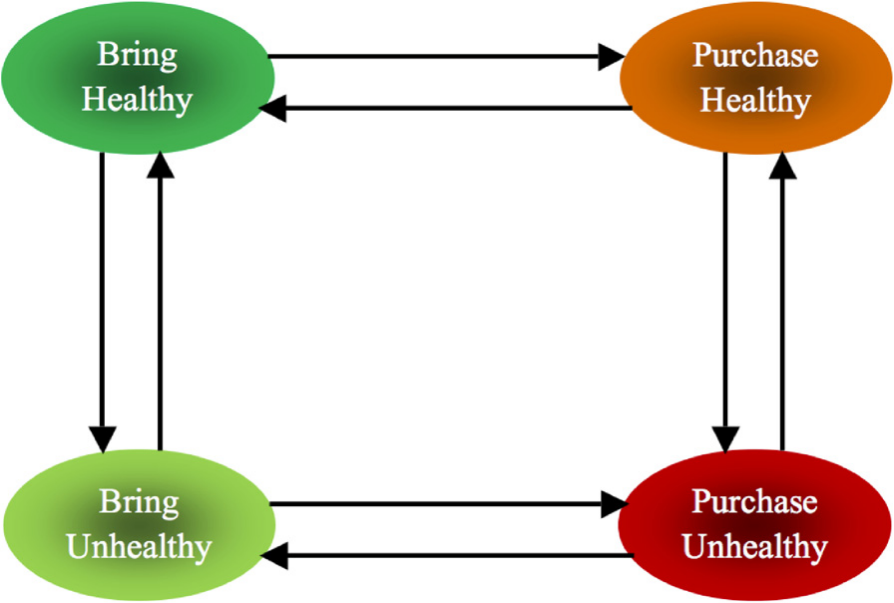
Model structure illustrating transition between individual states.

In Figure 1 we depict the potential state transitions present in the model. Influences do not have immediate effects, but accumulate over time. For example, if an individual who normally purchases healthy food interacted with individuals who bring healthy/unhealthy foods on a daily basis, the former might be influenced over time to begin purchasing food. Depending on the strength of the social influence exerted on the population (i.e., positive or negative) the individual might eventually transition between states of healthy or unhealthy food preference. In a case where a negative social influence is exerted on the population, the individual will be more inclined to bring unhealthy foods. The model assumed that there is no direct transition between *Bring Healthy* and *Purchase Unhealthy*, and *Bring Unhealthy* and *Purchase Healthy*. This is because of the gradual taste development for a new food which is affected by various factors such as: flavour learning, nutrient learning, exposure to new food and medicinal effects [33].

Social influences for bringing or purchasing healthy or unhealthy foods are tracked using two social counter associated with each student. For each student ‘*s*’ we denote the Bring/Purchase social counter at time ‘*t*’ by *BP*_*s*_(*t*) and the Healthy/Unhealthy social counter at time *t* by *HU*_*s*_(*t*). During each time step of the model these counters are increased or decreased based on the behaviour of each students neighbours.

Two types of social influences are considered in the model. First, a student can influence his or her classmates by discouraging or encouraging them to bring or purchase food. Second, students can also influence their classmates by discouraging or encouraging them to eat healthy or unhealthy foods. Social influences are accumulated over time from peers within the defined neighbourhood, affecting the states of neighbouring cells corresponding to parameter settings. The strength of the social influence regarding bringing or purchasing food is determined by the parameters *α*^*BH*^,*α*^*BU*^,*α*^*PH*^, and *α*^*PU*^. The strength of the social influence regarding eating healthy or unhealthy foods is determined by the parameter *β*^*BH*^,*β*^*BU*^,*β*^*PH*^, and *β*^*PU*^. These are conceptual parameters that capture a multitude of influences present in the environment. These influences can occur at the individual scale, such as influences of friends or parents on bringing or purchasing healthy or unhealthy foods, or at the environmental scales, such as influences of the type of foods in the cafeteria in school or food advertisements in TV. To summarize the whole model formally, we define two social counters which garner the social influences as:

(1) $$\begin{array}{l} BP_{s}(t)=BP_{s}(t-1)+\alpha^{\text{BH}}R_{s}^{\text{BH}}+\alpha^{\text{BU}}R_{s}^{\text{BU}}-\alpha^{\text{PH}}R_{s}^{\text{PH}}\\ \;-\alpha^{\text{PU}}R_{s}^{\text{PU}} \end{array}$$

(2) $$\begin{array}{l} HU_{s}(t)=HU_{s}(t-1)+\beta^{\text{BH}}R_{s}^{\text{BH}}-\beta^{\text{BU}}R_{s}^{\text{BU}}+\beta^{\text{PH}}R_{s}^{\text{PH}}\\ \;-\beta^{\text{PU}}R_{s}^{\text{PU}} \end{array}$$

where the parameters $\left(R_{s}^{\text{XX}}\right)$ are the number of neighbours of type ‘*XX*’ for the student ‘*s*’ in the Moore neighbourhood. For instance, for $\left(\mathrm{XX}=\mathrm{BH},R_{s}^{\text{BH}}\right)$ represents the number of students who bring healthy food to the school. At each time step, all cells in the lattice are updated simultaneously.

### Model update with purchasing power

Although, clearly, there exist many other factors that influence student eating habits, the influence that classmates have on each others eating behaviour is the most significant one [34]. As such, for this stage of modelling, only social influences are considered. Other influences might include the impact of teachers, educational curriculum, advertisements in the school’s environment and the type of food provided by school’s cafeteria, etc. Future work will examine how such influences could be incorporated into the model.

In this section we update the model with one extra external factor. In particular, some students may desire to purchase food, but their parents refuse to give them any money to do so. Such students are forced to bring food regardless of the *BP*_*s*_ value. For each student we introduce a parameter *PP*_*s*_∈{0,1}. If *PP*_*s*_=0 then the student is unable to purchase food, and always brings food. If *PP*_*s*_=1 then the student is able to purchase food, and the model treats the student as described above.

#### Transition Rules

This CA model attempts to replicate the social interaction between students within a social neighbourhood. In order to transition, an individual must follow the transition rules. We assume that at the initial state *BP*_*s*_ and *HU*_*s*_ are normally distributed with *σ*=0*.*5 and *μ*=0. These values are reset to 0 when a transition happens. For example, if a student that normally brings healthy food to school where general purchasing power among students is high, and they interact most frequently with those that purchase healthy food, they will eventually be influenced to purchase healthy food too. The transition may work inverse to that as well, with students whom normally bring unhealthy food being influenced to eventually purchase unhealthy food.

**Case I:***s* is a BH (Bring Healthy) 

• if $BP_{s}(t)<\Gamma_{l}^{\text{BP}}$ and *PP*_*s*_=1 then *s* becomes a PH (Purchase Healthy)

• if $HU_{s}(t)<\Gamma_{l}^{\text{HU}}$ then *s* becomes a BU (Bring Unhealthy)

**Case II:***s* is a PH (Purchase Healthy) 

• if $BP_{s}(t)>\Gamma_{u}^{\text{BP}}$ then *s* becomes a BH (Bring Healthy)

• if $HU_{s}(t)<\Gamma_{l}^{\text{HU}}$ then *s* becomes a PU (Purchase Unhealthy)

**Case III:***s* is a BU (Bring Unhealthy) 

• if $BP_{s}(t)<\Gamma_{l}^{\text{BP}}$ and *PP*_*s*_=1 then *s* becomes a PU (Purchase Unhealthy)

• if $HU_{s}(t)>\Gamma_{u}^{\text{HU}}$ then *s* becomes a BH (Bring Healthy)

**Case IV:***s* is a PU (Purchase Unhealthy) 

• if $BP_{s}(t)>\Gamma_{u}^{\text{BP}}$ then *s* becomes a BU (Bring Unhealthy)

• if $HU_{s}(t)>\Gamma_{u}^{\text{HU}}$ then *s* becomes a PH (Purchase Healthy)

where $\Gamma_{l}^{\text{BP}},\Gamma_{u}^{\text{BP}}$ are the lower and upper bound respectively for the *BP*_*s*_counter. Similarly, $\Gamma_{l}^{\text{HU}},\Gamma_{u}^{\text{HU}}$ are defined to control the healthy/unhealthy social behaviour counter of the model.

**Note:** The transition of state of a student from one state to another is based on social counters *BP*_*s*_ or *HU*_*s*_. If both counters reach the transition points simultaneously at any given instance of time, then transition can be arbitrarily selected. In our case, we give preference to *HU*_*s*_, as this study aims to model the healthy eating behaviour.

### Parameters settings

*Initial population:* In order to test the efficacy of the model, the school environment needs to be consistent and repeatable through all simulations, whereas the parameters (alpha, beta and purchasing power) are allowed to take on different values. This has been achieved by using a pseudo random number generator function to create the initial population sample. In essence, we are using the same initial eating pattern of student population across all simulations.

*Model parameters:* The *α*and *β* parameters are conceptual parameters which are assumed to have similar effect as that of probability value in any given system, so we restraint them as, *α*and *β*∈[0,1]. Moreover, these parameters exert competing forces in the whole system, so the model is sensitive to the difference between all positive and negative influences. For the transition rules, the lower and upper bounds of *Γ* are defined as −1 and + 1. These *Γ*values are checked against the social counters, using relational operators ‘<’ or ‘>’, to change the state of the student [35,36]. These well defined transitions rules ensure reproducibility of our results.

To simplify the model, we used a single variable to represent all positive influences as *P *= *α*^*BH *^= *α*^*BU *^= *β*^*BH *^= *β*^*PH*^ and all negative influences as *N *= *α*^*PH *^= *α*^*PU *^= *β*^*BU *^= *β*^*PU*^. One of the other possible choices for aggregation of positive influences can be $P\;=\;\frac{\alpha^{\text{BH}}+\alpha^{\text{BU}}+\beta^{\text{BH}}+\beta^{\text{PH}}}{4}$ and for negative influences $N=\frac{\alpha^{\text{PH}}+\alpha^{\text{PU}}+\beta^{\text{BU}}+\beta^{\text{PU}}}{4}$. Since the *Γ* values are within the range of −1 to + 1, so we assumed $\Gamma_{l}^{\text{BP}}=\Gamma_{l}^{\text{HU}}=-1$ and $\Gamma_{u}^{\text{BP}}=\Gamma_{u}^{\text{HU}}=+1$. The sensitivity of the model can also be defined as |*P*−*N*|. Based on these model specifications, we present results on different simulated environments in the next section.

## Simulation and results

Simulations examined how our CA model behaved under different parameter settings for social influences and purchasing power. Our first simulation showed how the population responded to an increase in purchasing power and positive social influence. Following this, we used a simple exercise to illustrate the effect that individual cells have on each other by gradually increasing the weight (*β*) associated with the healthy/unhealthy (HU) counter. The final simulation was a phase diagram that illustrates the precise social influence values that affect global trends in the population. As mentioned in the previous section, the parameters *BP*_*s*_ and *HU*_*s*_ may happen to reach the transition points simultaneously during simulations, so we arbitrarily give preference to *HU*_*s*_, as the main focus of this study is to model the healthy eating behaviour. Simulations were implemented using MATLAB 7.9.0 (R2009b). The Operating System is Windows 7 Service Pack 1 64-bit. The hardware configuration of the machine running the experiments is Intel CORE i5 2.30GHz with 4GB memory. The executable files or source code can be provided upon request.

### Impact of purchasing power and positive social influence

**Figure 2:**
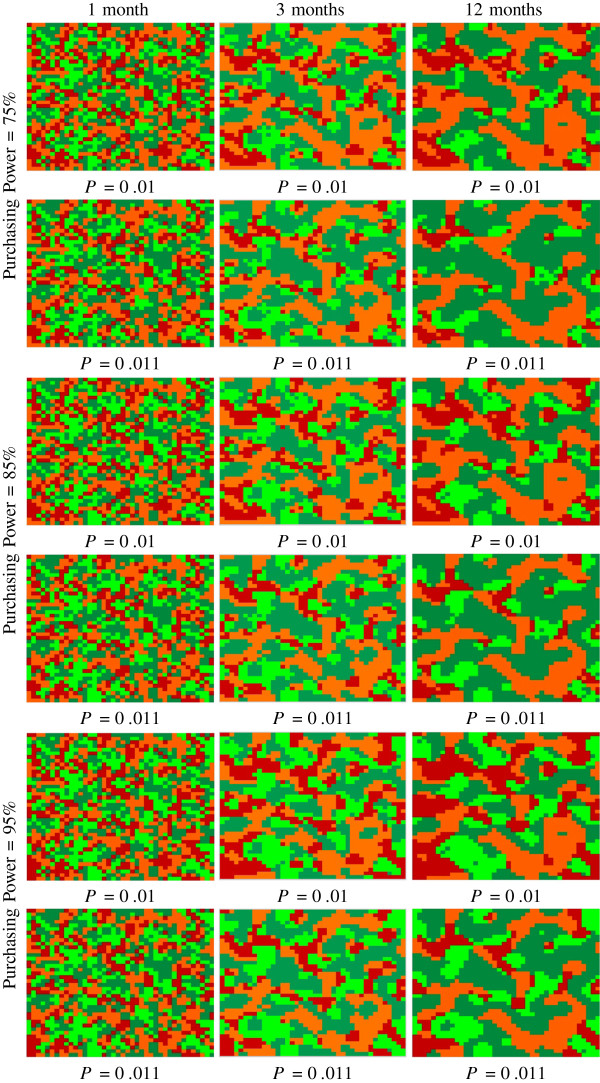
**Scenarios illustrating change in eating behaviour assuming increase in social influence and purchasing power.** Dark green color represents students who bring healthy food, light green represents students who purchase healthy food, orange color represents students who bring unhealthy food and red color represents students who purchase unhealthy food items. The size of the CA grid is fixed to 40×40, which defines the number of students. The parameter exerting negative influence, *N*=0*.*01 is constant across all simulations.

The columns of Figure 2 are the snapshots of CA simulation at different times with the start time of zero. The time-step is equivalent to a single CA iteration, therefore 30 iterations are approximately equal to 1 month, 90 iterations are equal to 3 months and so on. Rows represent different value settings of the parameter purchasing power (*PP*_*s*_), or the total portion of students with the ability (money) to purchase food. All students without purchasing power bring healthy and unhealthy food in the model and are assumed to be unable to purchase food throughout time steps. The initial population, say 100 students, is uniformly divided into four categories, 25 students in each group. The *PP*_*s*_=75*%*, allows all 50 students belonging to PH and PU categories to buy food from school. The remaining 25% of *PP*_*s*_ is shared among students from BH and BU categories (50 students). So, approximately, 25 students (50% of 50 students) cannot buy food from school, even if they wish so.

The positive social influence defined here is the influence from neighbours that bring food and having healthy food, as well as the culmination of other environmental factors. In the first row, both positive and negative social influences are the same, i.e., *P*=0*.*01,*N*=0*.*01 and the *PP*_*s*_ is kept at 75% level. Therefore, eventually, the numbers of students having healthy (dark green and green) and unhealthy (orange and red) food are similar. The reason there are more students bringing (dark green and orange) food than those of purchasing (green and red) is because 25% of students have no purchasing power and cannot change to either state of purchasing. As the positive social influence increases by 10% (0*.*01→0*.*011), an increased proportion of students start to bring healthy foods, as opposed to bringing unhealthy. Likewise, there is an observed decrease in visual clustering of students purchasing and bringing unhealthy foods. This pattern is most likely due to the impact that a positive eating influence can exert on a population where only 75% percent of students have *PP*_*s*_.

In the scenario where the population has an 85% *PP*_*s*_, similar overall clustering trends are observed when compared to the 75% *PP*_*s*_scenario. Under these parameters there is an expected dispersive pattern, with eating behaviour states gradually forming clusters with neighbours - i.e., PU and BU individuals clustering together. However, in this scenario, the overall distribution appears to be more uniform than at 75% *PP*_*s*_. This model behaviour is expected, as an increase of 10% *PP*_*s*_would enable individuals belonging to states PU and PH to purchase more food, resulting in an increase of individuals transitioning to these states. When positive healthy influence is increased by 10% (0*.*01→0*.*011), again there is visual clustering of both purchasing and bringing states. However, clustering among individuals who bring and purchase healthy foods have replaced individuals who bring and purchase unhealthy foods, and an increase of individuals purchasing healthy foods is a an emerging trend in the population.

In the final scenario, the population has an overall *PP*_*s*_of 95%. Assuming positive and negative social influences to be equal (*P*=0*.*01,*N*=0*.*01), the overall trends remains consistent over time; states of bringing and purchasing tend to cluster. After 12 months, individual states appear to be uniform, with a marginal increase in clustering among individuals that purchase food. This trend is consistent with the similar observation assuming a population with 85% *PP*_*s*_, whereby more individuals are enabled to purchase food. After increasing the positive social influence by 10%, individuals appear to cluster more prominently towards bringing and purchasing healthy foods. Although more purchasing of unhealthy foods is observed, there is also a marked increase of individuals purchasing healthy foods. Assuming an equal population distribution, this model behaviour suggests that if there are positive social influences in schools and surrounding environments, individuals’ choices will gravitate to healthy behaviours, regardless of purchasing power.

In general, there is one common characteristic of changes across time; all colored states are dispersive initially, but eventually form distinct clusters among similar, neighbouring states. This is consistent with the findings that students’ eating behaviours tend to form clusters with individuals closest to them in a social network.

### Impact of social influence on healthy eating

**Figure 3:**
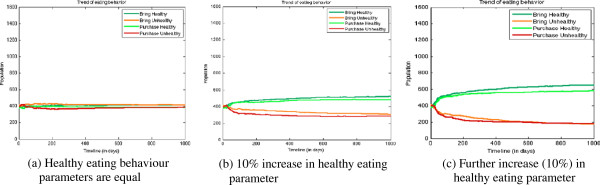
**Scenarios illustrating change in eating behaviour pattern due to increase in social counter parameters, healthy eating behaviour.** In Figure **(a)**, *P*=*N*=0*.*01. Eating behaviour of students is uniformly distributed among all categories (i.e., 25% of 1600=400). But when we increase the healthy eating influence by 10%, i.e., *β*^*BH*^=*β*^*PH*^=0*.*011 in Equation 2, then we observe positive transition, Figure **(b)**. Same trend is further observed if we assume the values of *β*^*BH*^=*β*^*PH*^=0*.*012, Figure **(c).**

This simulation changes positive HU influence level (*β*^*BH*^and *β*^*PH*^in Equation 2) only, and assumes all individuals have the potential to buy food, or 100% *PP*_*s*_. This influence is different from the social influence parameter in that only the cells denoted as healthy or unhealthy affect neighbouring states. Since there are no other factors affecting the choice to bring or purchase, except for *PP*_*s*_(they are equal in this scenario), the entire population of the four states are close and exhibit similar behaviours. In Figure 3a, the influence parameters for Healthy or Unhealthy are equal. The population of each group stably remains similar with 25% each as their initial portion. In Figure 3b the positive parameters of HU counter are increased by 10%, illustrating the clustering tendencies of healthy choices also exhibited in the previous section. With another 10% increase (Figure 3c), there is further increase in healthy eating behaviour. This simulation is illustrative of the parameterization of cell transition rules. Other combinations of parameter values are possible, but were not included in this analysis for presentation reasons.

**Figure 4:**
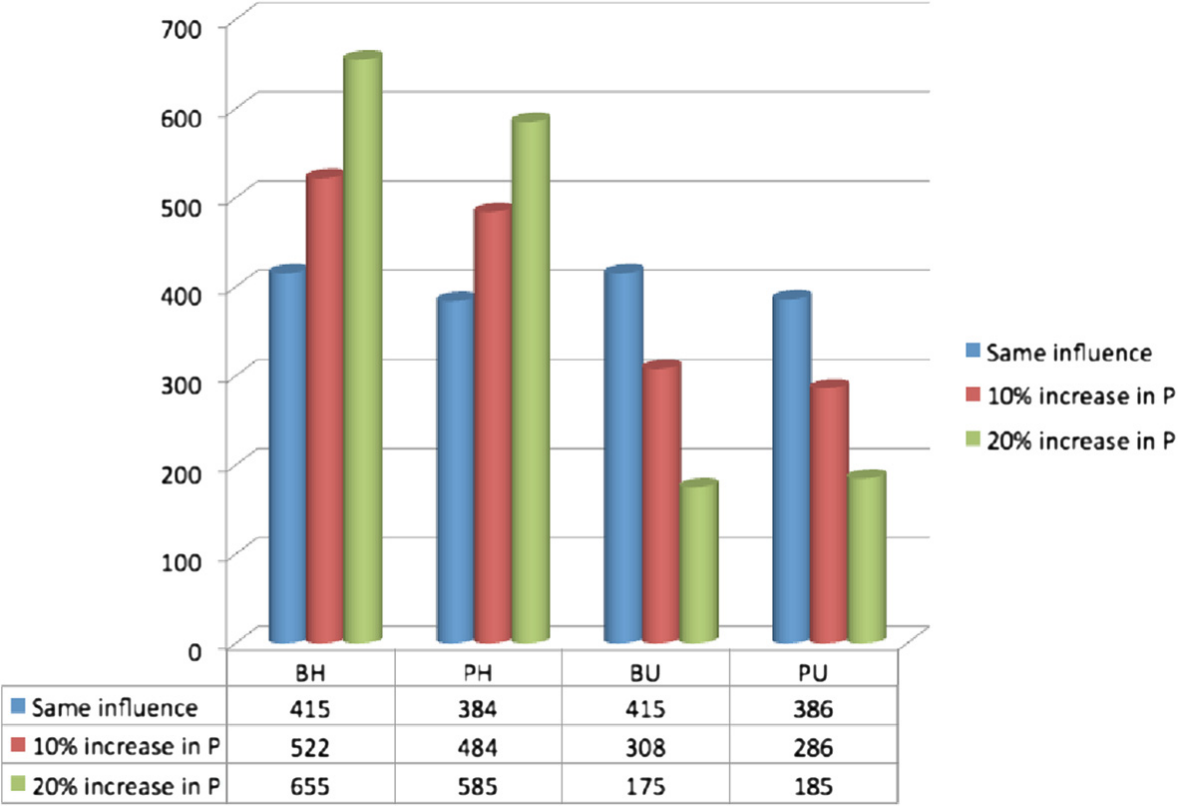
**Statistical information of eating behaviour patterns when we change the positive influence in parameter settings.** The blue colored bars represent the results of first simulation run when all parameters are same. The red and green colored bars represent the scenarios when we increase the values of positive parameters.

The statistical information observed at the end of simulation runs of Figure 3 is summarized in Figure 4. It is important to note that all these simulations have same initial population distribution but different parameter settings. More specifically, we have randomly distributed 1600 (sum of row values at the bottom of Figure 4) students in four eating behaviour states. This figure depicts that when we run the simulations for 1000 days with same initial influences (*P*=0*.*01,*N*=0*.*01), the students’ eating pattern is approximately evenly distributed. But when we increase the positive influence parameters of HU counter, we observe a positive transition in eating patterns, i.e., eating health.

### Phase diagram: Transition between healthy and unhealthy eating

**Figure 5:**
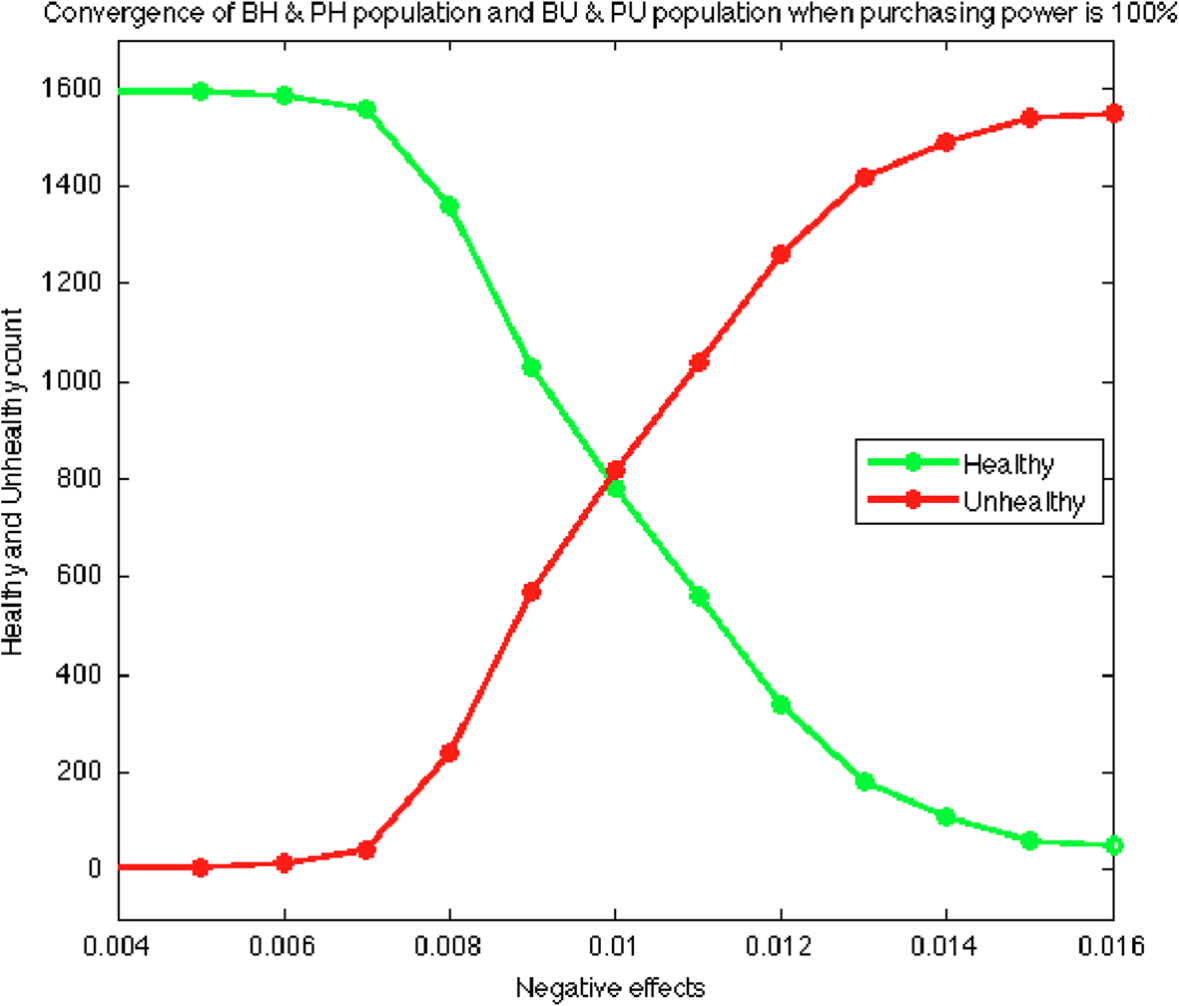
**The Phase diagram depicts the overall transition of healthy and unhealthy eating patterns among students.** This has been constructed by varying the negative influence on the horizontal axis while keeping the positive influence at a constant level.

This experiment provides a global depiction of how variables behave under particular parameter values and positive/negative impact. The phase diagram presented here illustrates that the system eventually evolves into either one of two phases when it is stable: Healthy phase or Unhealthy phase. A Healthy phase means that the stable population of Healthy (BH and PH) is larger than that of Unhealthy (BU and PU), and vice versa. We have known from a previous experiment that the change of the positive or negative impact level can change the final dominant population. It implies that there exists a critical point for positive/negative impact such that the system is stable at neither Healthy phase nor Unhealthy phase. Figure 5 depicts the stable counts of Healthy/Unhealthy population versus different value of negative influence with a fixed positive impact level at 0.01. The critical point for the negative impact level is approximately at 0.01, and the transition is non-linear. The largest acceleration is around the critical point. This suggests that at certain point, smaller efforts to modify positive/negative impact of environment has the potential to yield more healthy eating behaviours from students.

## Discussion

This exploratory CA model illustrates how a robust mathematical modelling approach can be applied to analyze non-linear relationships associated with high-school student’s social interactions and eating behaviour. Our results suggest that when a positive environmental influence is introduced to individuals, whether manifest in inter-personal relationships and friendship ties, school health promotion initiatives, positive parental influence, reduction of competitive foods in school, or other environmental and structural changes, students will show a higher propensity to choose healthy eating habits.

The simulation illustrating the gradual increase of purchasing power and positive environmental influence represents hypothetical scenarios of the potential dynamic processes that interplay with adolescent eating behaviour and environmental influences. This simulation shows that when positive and negative environmental influences are set to the same level of *P*=0*.*01, and *PP*_*s*_ is gradually increased, students bring both healthy and unhealthy foods remains constant, and students purchasing healthy and unhealthy foods steadily increases. This suggests that adolescent students will purchase both healthy and unhealthy food when present, and when the population’s overall purchasing power permits it. Though past studies show support for students purchasing unhealthy food when it is readily available [37,38] evidence supporting increased student purchasing of healthy foods is mixed [39-41]. As the positive social influence parameter was increased by ten percent to *P*=0*.*011, students previously purchasing unhealthy foods, transitioned to a bringing healthy state. Such a change in the system suggests that the positive social influence can encourage students to bring healthy foods from home. An example of this social phenomenon can be seen from a study that implemented a school health promotion strategy that paired up school-aged students from different grades, and allotting daily class time for instruction on healthy-living and nutrition [42]. Students in the intervention group showed an increase in healthy-living knowledge, behaviour and attitude, and lower blood pressure measurements than the control group. It is conceivable that a change in eating behaviour may take shape through other health-oriented activities, such as physical activity [43]. The social influence parameter may also represent the influence of other elements in the adolescent’s exogenous and endogenous environments. Social influence could also be in the form of school food program policy changes, or neighbourhood level influences such as increased accessibility to healthy foods. Future simulations may also be tailored to include multiple environmental parameters, often used in dynamic systems models, such as agent-based models.

Isolating parameters of models allowed us to experiment with different pathways of eating influence (Figures 3a, 3b, 3c). When the parameter representing transition rules between healthy and unhealthy states was increased, this change was exerted on neighbouring cells and their states changed accordingly. Our simulations in Figures 3a, 3b and 3c illustrate this effect by setting all parameters equal, except for HU and running the model over 1000 time steps. As time increased, eating behaviours clustered based on the individual’s state of healthy or unhealthy. As the healthy/uhealthy parameter gradually increased, clustering behaviours persisted, and the gap between the healthy and unhealthy populations widened. Results from the simulation show resemblance to other studies that examined the effect of social behaviour on physical activity [43], and obesity [23,24,44]. The social network behaviour observed in these studies posited that individuals exhibiting similar interests, behaviours, or traits, will ultimately form groups with others of the same distinction. The parameter settings for this simulation were specifically tailored to illustrate robustness of the model. Conversely, parameter settings could be altered to reflect an increase of influence from all individuals in the bring/purchase category.

Phase diagrams are another way to monitor how individual processes interact with each other in dynamic models. As with the previous simulations in diagrams (Figure 3a, 3b, 3c), the phase diagram isolated parameters to show how the system interacted under individual parameter permutations. Our phase diagram simulation suggests that at a certain point, the positive environmental influence ceases to insulate healthy students from adopting unhealthy behaviours, and the negative environmental influence increases the amount of unhealthy student behaviour exponentially. The use of phase diagrams in CA modelling has been used elsewhere in the literature [35,36], and is supported as an effective utility for understanding global behaviours in dynamic models.

### Adolescent/Childhood obesity

School-aged children and adolescents are a population who view the environment through a highly impressionable lens. The complexities of the environments they interact with interdependently affect the way their behaviours are formed and continuously adapted. Forming a better understanding of how environments interact with one another requires a holistic interpretation of individual pathways and mechanisms as dynamically linked parts of a whole. Our model suggests that, as individuals who exhibit similar behaviours socially interact, their ties grow stronger and produce a pulling effect on proximal actors in their social environment. When a change to the population’s environment takes place through the increase in the environmental parameter, the model suggests individuals will react to the respective change and their behaviours will change according to their social proximity and initial state. As research in social behaviour and health increases, the need for richer datasets will prove ever important if we are to build a better understanding of the underlying mechanisms. Illustrative of the potential for mathematical modelling as a means to answer these questions is the study from Bahr and colleagues [24], which simulated interventions in the population by implementing different parameter settings. Similarly, our model applies a dynamic systems modelling approach to examine a social interaction and eating behaviour, two pathways that are suspected to influence individuals consumption of food. The parameters of the model may represent a number of variables in the environment. Likewise, the environmental parameters we used in this example were meant to represent a myriad of factors occurring at multiple levels. While we know this to be true of obesogenic environments, we acknowledge that such an assumption is not likely to hold true for all populations. Rather, the intended use of the social influence parameter should be to replicate different environmental factors or interventions as single influences affecting the entire population. Examining how the population is affected by the newly introduced influences will aid with optimization of parameters and addition of more complexity. This model should serve as an impetus to refine data collection methods to be more suitable for models of this nature.

Our model does have limitations. This is the initial stage of model development, and as every developing model requires, real data is needed in order to calibrate parameter settings. Model assumptions are commonplace in mathematical modelling and as such, our model strived for simplicity in our assumptions in order to illustrate the robustness of this approach. Future implementations may include different parameters that emulate environmental influences. For instance, time one spends in school is not the only socializing that individuals partake in, and individuals may strengthen bonds in extracurricular school and non-school activities. Thus, having a more complete picture of an individual’s socialization restraints and enablers, as well as their existing social network, can help calibrate the model to a more realistic depiction [26]) Individual characteristics were not included for each individual as real data was not accessible to us. It is likely that eating norms and socialization vary along ethnic lines. Socio-economic status may also be a predictor of an individual’s food quality brought to school, or the individual’s ability to purchase food at school. As well, an individual’s weight status will also explain part of the eating behaviours inherent in an individual. Incorporating both contextual, and individual determinants in to model development will greatly enhance calibration of future models.

## Conclusion

Conceptualizing the social environment of individuals is a crucial step to increasing our understanding of obesogenic environments of high-school students, and moreover, the general population. Viewing environments as independent from one another ignores feedback loops between exogenous factors, and potentially obfuscates the underlying mechanisms for certain outcomes. The results of this exploratory exercise demonstrates that students will cluster based on their preference for healthy and unhealthy food. When a positive environmental influence parameter was introduced, positive effects to the overall population were experienced, resulting in more students making healthy decisions. Calibrating this model by using real data as inputs will dramatically increase the potential for experimentation assuming a variety of environmental parameters. Validation of our model may also culminate in greater knowledge transfer between research disciplines and public health professionals. Parameters may also be included in the model to replicate policy implementations, such as the removal of vending machines in school, represented as a positive influence parameter. This feature of our approach would actually serve as policy evaluator tool as well. Future research intending to adopt this approach should aim to effectively communicate to stakeholders and disciplines the benefits as outlined above, as innovation of our method is dependent on their support and research ingenuity. A cellular automata approach to analyzing multilevel, interdependent factors in health research should be considered a novel tool for improving knowledge about public health intervention strategies.

## Competing interests

The authors declare that they have no competing interests.

## Author’s contributions

VD and AA conceived the idea and formulated mathematical model. VKM and TW implemented the model and CF wrote the paper along with AA. VD and VKM analyzed the simulations. All authors critically reviewed the manuscript and read and approved the final version.

## Pre-publication history

The pre-publication history for this paper can be accessed here:

http://www.biomedcentral.com/1471-2288/12/155/prepub
